# "The Hospital" Nursing Mirror

**Published:** 1897-10-02

**Authors:** 


"The Hospital" Nursing Mirror.

Being the Nursing Section of "The Hospital."

[Contributions for this Section of "The Hospital" should be addressed to the Editor, The Hospital, 28 & 29, Southampton Street, Strand, London, W.C., and should have the word "Nursing" plainly written in left-hand top corner of the envelope.]

### News from the Nursing World.

### THE LONDON HOMŒOPATHIC HOSPITAL.

The new Wing of the Nurses' Home at the Homœopathic Hospital is making rapid strides towards completion. The builders should be out of it by Christmas, after which furnishing and readjustment will commence. The enlargement consists principally of bath-room, lavatories, a few bed-rooms, and offices. There is to be a nice dining-room at the top of the building on a line with the hospital kitchen, from which the meals will be served. The sitting-room and library will be on the ground floor. Covered bridges join the home to the hospital. The site at command of the hospital authorities is very limited, and although they have devoted as much of it as possible to the nurses, the accommodation will still not be sufficient to house the whole of the hospital and private staff of nurses.

### THE FRENCH PRESIDENT AND THE HOSPITALS.

Ever since his occupation of the post of President of the French Republic, Mr. Felix Faure has never failed to devote some of his well-filled time to visiting and assisting the sick in hospitals. He has established an association for clothing poor patients on quitting the hospitals, and when at the Nanterre Hospital recently, he contributed £40 for the purpose. Some of the tradesmen willingly co-operated in the kindly act by providing clothing at a reduced rate. At the first distribution the women were completely clothed for 15s. each, and the men for 24s.

### WANTED, VOLUNTEERS.

Over nine hundred cases of typhoid fever have been reported at Maidstone since the outbreak, and, as well can be imagined, the resources of the district as to nurses are totally exhausted, and the chief of the local medical staff corps has issued a printed circular asking for more volunteers to assist in nursing at night. It is unnecessary to point out to nurses how much depends on the nursing of typhoid. Maidstone is therefore in urgent need at the present time, and were such an organisation as was recently started in aid of a foreign country to be formed to supply and defray the expenses of trained nurses for the poorer sufferers at Maidstone, there would doubtless be found plenty of work to be done. Volunteers should tender their services to Dr. Poole, superintendent medical officer during the epidemic, Maidstone; or to the Chief of the Local Medical Staff Corps, care of the Mayor, Maidstone, Kent.

### THE ROYAL BRITISH NURSES' ASSOCIATION.

It is announced that a public meeting will take place in St. Martin's Town Hall, Charing Cross, on Wednesday, October 13th, at four p.m., "under the auspices of the 'Members' Rights Defence Committee of the Royal British Nurses' Association,' when the reasons for the public inquiry which is now being demanded into the management of the Royal British Nurses' Association will be explained, and important resolutions will be proposed." It would seem to be desirable in the public interest that some representatives of the Royal British Nurses' Association should attend this meeting to secure that the actual facts of the present situation shall be correctly stated for the guidance of the Press and the public. It would be interesting to know who constitute the so-called "Rights Defence Committee," whatever that may be?

### THE NEW NURSES' HOME, DUNDEE.

Great pains are being taken to render the new nurses' home at the Royal Infirmary, Dundee, comfortable and attractive. There are to be 37 bed-rooms, the walls of which are covered with pretty and cheerful paper. The woodwork is lightly grained, the floors being margined with bog oak. In the bath-rooms the woodwork is of pitch pine, and the walls and flooring of these are tiled. In the drawing-room grained walnut woodwork is used; the ceiling is panelled, and decorated with colourings to match the paper. There is a sitting-room and a library besides this apartment decorated in a similar manner. The home is the gift of Sir William Ogilvy Dalgleish, and will add immensely to the comfort of the nurses at the Royal Infirmary.

### BEADNELL NURSING ASSOCIATION.

The first annual report of the Beadnell and District Nursing Association, which was founded in 1896, has proved it to be a most useful institution. The nurses serve a population of over 5,000; three are at work at present and two others are training at Plaistow, and will shortly be employed. One of these new nurses has received one of the North Cumberland County Council's scholarships, through which means her training has been provided. The work of the nurses has been so much appreciated and found so necessary that in some cases extra help has been obtained.

### OPHTHALMIA IN THE NEWLY-BORN.

The public sanitary authorities in France have issued some useful instructions to midwives concerning ophthalmia neonatorum. With the instructions are printed directions how to detect the symptoms of the disease, and also as to preventive and precautionary measures to be taken.

### A GOOD EXAMPLE.

More than once we have had to speak in very strong terms of the arbitrary and harsh conduct of guardians in keeping to the letter of the law in respect to the remuneration of nurses. We are glad to be able to give an instance of another kind in regard to the Dover Board of Guardians, who allowed an old nurse, too feeble to continue her work, to retire, fixing her term of service to the requisite number of years in order that she might obtain her retiring allowance of £26 per annum.

### "PROFESSIONAL" PARTIES.

THE Arthy Guardians recently advertised locally for a night nurse, and received no applications for the post. The reason was discussed at a meeting subsequently, and the Chairman explained that the regulation placing the nurse under the supervision of the medical officer and the nuns was the probable objection. A Guardian suggested that the "parties look upon themselves as professionals," and that they might object to any but the doctor over them. The Clerk remarked that the appointment of night nurses had become a craze all over the country, and he supposed they were all engaged.

### THE NURSES' HOME FOR HULL.

THE scheme to provide a district nurses' home for Hull as a Jubilee commemoration offering has not progressed very vigorously. It appears to us to be a happy arrangement to amalgamate the Jubilee scheme with the existing District Nursing Association, as the expenses of administration will thus be minimised. The subscription list does not amount, however, to £3,000, and to establish a reasonable number of nurses for the poor of so large a population as Hull £5,000 at least is estimated to be necessary.

### LONG HOURS AT BRISTOL.

AT a recent meeting of the Bristol Board of Guardians a statement was made that the nurses were on duty for thirteen hours a day. A motion asking for the appointment of a committee to inquire into the case was put and seconded. The motion was lost, however, the Chairman remarking that it was not long ago that a committee considered the whole subject.

### AMBULANCE WORK AT BERLIN.

FROM October 1st every hospital in Berlin will be connected with the new Ambulance Society instituted under the presidency of Professor von Bergmann.

### ASYLUM NURSING.

AT the Richmond Lunatic Asylum, Dublin, an attendant has just been brought before the Court for having broken the arm of a patient by using undue force. Dr. Eustace proposed that he should be dismissed, but the alderman before whom the nurse was brought thought that a fine of £1 and a reduction in rank to the bottom of the staff of nurses would be sufficient punishment. With all due regard to the extreme difficulty and the aggravations which attendants on the insane must of necessity undergo, we cannot but think that for the sake of discipline and for the good of the patients Dr. Eustace's recommendation should have been followed. The insane are peculiarly dependent upon those who have charge of them. The status of asylum attendants both in England and Scotland is showing signs of improvement, but owing to the conditions of the service and to the reputation in which they are generally held, the class from which these attendants is drawn is for the most part inferior, and it cannot be too strongly insisted upon that kindness shall be exercised towards patients, and that violence is both reprehensible and unnecessary. The medical attendant is only occasionally able to witness the treatment to which the inmates of an asylum are subjected by the attendants, and the testimony of the patients themselves is too unreliable to be depended upon. Therefore a reliable staff is absolutely essential.

### NURSING AMONGST LEPERS.

A CORRESPONDENT, who has spent some years in attendance upon lepers, thinks that some of our readers who may contemplate nursing this class of patient may find it of interest to learn some of her observations. She finds that one of the chief difficulties to overcome in dealing with lepers is their dislike of cleanliness. Lepers avoid a bath with persistency, and prefer a daily wipe with a damp rag. They have a tendency to collect and hoard dirty articles and clothes, and have to be watched in this respect. They also show a fondness for rotten fruit. In fact, a general tendency to squalor and slovenliness is characteristic. From her own observation, our correspondent notes that with cleanliness is associated an improvement in the diseased person, and she advocates its enforcement by all who have to do with lepers. She says those who are separated totally from others, as at Robben Island, have a far better chance of happiness than in small, isolated groups only slightly removed from their fellow creatures.

### NURSES FOR SOUTH AFRICA.

NEW openings for trained nurses present themselves frequently. One of the latest appeals for their services was on the part of the South African bishops attending the Lambeth Conference. As in the case of all nursing abroad a great many drawbacks attend the position. The engagements are for three years, which is a long time if the duties and surroundings prove distasteful, and a short time for which to sacrifice the chance of work at home when the nurse returns. Those who go out should make full inquiries, and then be prepared to continue the work for a considerably longer period than three years, if they need to think of pecuniary advantages.

### THE RED CROSS SOCIETY.

THE German Red Cross Nursing Society holds its annual meeting at Darmstadt on October 1st. Some very useful topics will be introduced for discussion, one of these being "United Help in Time of War and in Epidemics." The recent necessity for nurses in Greece has brought the subject especially forward at the present time, and there is undoubtedly a useful work before the Red Cross Society to organise a body of nurses ready to serve when necessary and from which suitable volunteers could be drawn. A knowledge of more than one language should be a necessary qualification. It is to be hoped that the subject will be more widely discussed at the International Congress to be held at Vienna.

### DISTRICT NURSES AT HUDDERSFIELD.

A HOME for district nurses has been established at Huddersfield, in commemoration of the Jubilee. Two nurses have been engaged, and the home is now being furnished. The sum subscribed for the purpose amounts to £2,890.

### SHORT ITEMS.

THE annual harvest festival and thanksgiving services were held at the Oriolet Hospital, Loughton, last week. Lady Gwendolen Herbert addressed the nurses, after which tea was served in the matron's room.

### Lectures to Surgical Nurses.

By H. A. LATIMER, M.D. (Dunelm), M.R.C.S. of Eng., L.S.A. of London, Consulting Surgeon, Swansea Hospital; President of the Swansea Medical Society; Lecturer and Examiner of the St. John Ambulance Association, &c.

### XV.—SEPTICÆMIA — PYÆMIA — HYDROPHOBIA — GLANDERS—TETANUS.

Now let us consider two other surgical fevers due to the operations of infectious germs—septicæmia (or septic infection) and pyæmia. I have grouped them together, for, although they are distinct diseases, they both owe their origin to the ravages wrought by micro-organisms, and the former sometimes is followed by the latter. In the last lecture I spoke of sapræmia, or septic poisoning, explaining that it was due to the absorption into the blood of the products of wounds. These products are of a chemical nature, and are produced in wound discharges by the action of bacteria or micro-organisms. The bacteria themselves do not get into the blood, but a poison formed by them does. In septicæmia the bacteria do gain admission, and, having done so, take on a fresh lease of life, increase in number, and evolve their poison there. I pointed out to you that the effects produced in sapræmia responded to the amount of the poison absorbed. I now draw your attention to the fact, which you will already have grasped, that in septicæmia the reverse obtains, and that, given the fact of a small number of germs having been absorbed, a great amount of disease will be caused owing to their multiplication in the blood. When this septic infection has taken place it reveals itself by a sudden rise of temperature, and during the whole period of disease the body heat varies greatly from time to time. Vomiting and diarrhœa also occur, and the patient rapidly wastes in flesh from the general disturbance in the body which follows. But, whilst the case remains as a true one of septicæmia, no abscesses in the body form. This marks the difference between it and pyæmia, a disease which owes its name to a belief by the older investigators that pus itself had gained admission into the blood; for in pyæmia, whilst we have all the constitutional symptoms which mark septicæmia, we have the additional one of the sudden formation of abscesses in different parts of the body—in the lungs, muscles, liver, spleen, and joints. The abscesses occur quite suddenly and are often indicated by the occurrence of rigors, and, as you would expect, the sufferer from pyæmia is in great danger of losing his life in consequence of the destruction they effect in important organs, as well as from the violent fever which prevails. The symptoms are brought about in this way. Micro-organisms—probably special forms due to previous cases of a like kind—develop in wounds; these find their way into the veins, and the blood present in these vessels then clots; the clots, which contain living organisms, break down into little bits; these infective bits are washed off and carried into the stream of blood which is returning to the right side of the heart; from here they are carried into the lungs, and if they are of moderate size one or more of them block up vessels there, and, wherever they stop, being living and infective, they cause a violent inflammation, which runs on into the formation of an abscess. Should the bits be very minute, and small enough to pass through the blood vessels in the lungs, they are carried into the heart, and, passing through that organ, are carried out by the arteries and are conveyed into the general circulation of the blood, when in some place or other they are arrested. Wherever one stops it does as I have just told you, inflames the part and sets up an abscess. From this you will see that while pyæmia is prevailing you never know when a new abscess will make its appearance, or what organ may be affected. It is peculiarly liable to occur where disease is affecting parts which are rigid and unyielding, where veins which have been opened by ulceration in soft parts, or caries or necrosis in bone cannot collapse, and so are kept open for the entrance of poisonous materials; and it is very infectious to other patients ill in a ward where the original sufferer lies.

As far as treatment is concerned, when these diseases occur, little more can be done than to attend to the patient's health by looking well to the wound of origin, opening abscesses quickly, should they be accessible, and by sustaining the sufferer by good and nourishing foods of a suitable kind, in the hope that he may be able to live through the storm which has arisen. As nurses, you will be concerned in the latter part of the treatment—in combatting the rigors which occur from time to time, in dealing with the violent sweatings which take place, and in watching carefully for any swellings, redness, and pain in different parts, which herald in new formations of matter. You will have to look out for bed-sores and retention of urine, and will find that it is advisable for the prevention of the former to place the patient on a water-bed; and you will take note of new symptoms which may arise, reporting them to the surgeon in charge at once, and keeping any suspicious evacuations which may have been brought away for his inspection. Thus, for instance, should a blockage occur in one of the kidneys the patient may have pain in the loin, and will want to pass water often, and the water may contain blood; should the same have occurred in the lungs there will be difficulty in breathing, cough, and, possibly, spitting of blood. Such symptoms as these, when noted by you and reported to the surgeon, will redound to your credit, and prove you to be intelligent observers, and valuable helps alike to him and to the patient.

I shall not dwell long upon those affections known as glanders, hydrophobia, and tetanus, as little will be gained by dealing fully with them, owing to the fact that they occur but seldom, and that they do not call for any exhaustive description on my part. Glanders and hydrophobia are strictly contagious diseases communicated from animals to human beings through infected discharges. In the first-named disease, discharges from the mouth, nose, or from abscesses gain admission to man through cracks or wounds in the skin, or from absorption by the mucous membranes (that is, inner skin) of the eyes, nose, and mouth; in the second case the infected saliva of the dog, cat, wolf, or other such animal who is suffering from rabies or hydrophobia, poisons the wound caused in a man during the time that he is being bitten by the animal. They are both very deadly diseases, and little can be done for the patients suffering from them. It is well to remember that those who suffer from them are infective to others by the contagiousness of their discharges; so if you ever have the task of nursing such cases, you will be careful to observe due precautions in this respect. Tetanus you will more often see, for, from time to time, cases present themselves in hospitals. The modern theory is that it is a disease due to the action of a special micro-organism, but many observers still hold that it may be set up by any great irritation to a nerve connected with a wound. Whatever may be its cause, the symptoms resulting from the disease are unmistakable when they occur. The first one, the one from which its ordinary name of lockjaw is derived, is that the sufferer complains of a stiff neck, and that he cannot open his mouth freely; quickly after this initial symptom violent cramps and distortions of different parts of the body set in, temperature rises, and, as the sufferer remains conscious, great agony is endured. If you ever have to nurse a case of tetanus, remember that the cramps or convulsions are set up by the slightest irritation to any of the senses in the suffering person—a draught of air, the "banging" of a door, a bright light, the movement of the bedclothes, sufficing to bring them on. The symptoms of hydrophobia are very akin to those of tetanus, but they differ from it in the history of the cause, the extraordinary dread of drinking (convulsions resulting from the mere sight of water), and in the delirium which accompanies the illness.

### Post-Graduate Clinics for Nurses.

By a Trained Nurse.

### XXXII.—DIETETIC IDIOSYNCRASIES.

Full allowance must be made, in supplying the dietetic needs of our patients, for individual idiosyncrasies and "fads" about food.

Many, or most illnesses are complicated by gastric difficulties, and in a nursing career we are likely to meet with so many varieties of digestive disturbance that we ought to realise as an accepted axiom that the food most easily digested by one stomach may prove a gastric barrier insuperable by another. Generalisation about most subjects is apt to be inaccurate. But so far as digestive fitness goes one will get hopelessly wrong by starting on the assumption that because Mr. A managed such and such a food well in spite of a "weak digestion," Mr. B, whose gastric energy is also weak, will do well on the same diet; because, most probably, the two cases will have no relation one to the other and cannot, therefore, be compared. As a general rule, each patient's digestion stands on its own merits, and each stomach has its own individuality, and must be considered from its own point of view. It does not belong to a type or class capable of being "lumped together" as able to dispose of certain foods.

It seems to me that it is just on this rock of generalities that so many nursing and food books split. For, taking up many such books at haphazard, we see statements such as these: "One pint of milk contains as much nourishment as two quarts of beef-tea." Now, without going into the questiod of the quality of the milk as compared with the quality of the beef-tea—and it will be seen that this important fact, which would entirely alter the accuracy of the statement, is entirely overlooked—a statement such as this is very misleading.

Even though such a comparison might be worked out as chemically correct, although this is extremely unlikely, the proposition does not stand for one moment. For you get a totally different effect on the digestion and the constitution from beef-tea to that which you would expect from milk. Added to this, there is the eternal truth that diet is by no means a question of chemical equivalents. For, were this so, it would be quite easy, and would save much trouble, were we to feed our invalids direct from the laboratory or chemist's shop. But we know—though it would be possible to supply the given proportion of chemical constituents necessary to human life—the patient would starve. For in food there is a mysterious quality and a natural combination of constituents which no chemist can imitate.

In some large text-books on nursing I have measured just three inches of space devoted to the subject of sick diet and the manner in which food should be prepared and served to the invalid. In other text-books no mention whatsoever of food is made throughout the various chapters devoted to the care of the sick. Thus one of the most material points in nursing is left out of account. Again that old, old statement which confronts us on some page of nearly every book on food as yet produced, that "eggs are as nourishing as meat," has no foundation, either in science or common sense. For the two foods are so different that it does not seem worth while to establish so misleading a comparison. Many sick persons may be able to take eggs when they could not take meat. And again, we know that many people—in health and sickness alike—are quite unable to take eggs, these having in some people a mysterious tendency to cause extreme biliousness and other ills. Altogether, and especially in matters of diet, I would ask my nurse readers to avoid the unfortunate habit of "lumping things together" in their own minds. A good many young nurses acquire a habit of remembering things in formula shape. Not having themselves that experience which comes only from long years of practice, they are apt to take general rules as their guiding stars. Lately I came across a young nurse in private practice who had the mania for digestive comparisons. She had read somewhere that "boiled rice is digested in one hour, while boiled chicken takes an hour and a half." The patient under her care had a weak digestion of chronic standing, and on all occasions, in and out of season, this young nurse set boiled rice before the luckless man, on the plea of its digestibility, quite leaving out of account the obvious fact which the book had not mentioned, that the two foods, both good in their particular way, serve totally opposite purposes.

The patient was being starved—at least so far as he would allow himself to be—in two senses: his stomach was starved by the insufficiency of nourishment afforded by the rice, and his palate was starved by the flavourlessness and sameness of the perennial dish.

And added to this unfortunate outcome of generalisation the statement was obviously and foolishly inaccurate. For there are large numbers of people who could not digest boiled rice in an hour just as many persons would not dispose of boiled chicken in ninety minutes.

Other statements in some types of nursing literature are equally unfortunate and misleading. For instance, one very common recommendation of this character is found in the advice that some varieties of fish are specially desirable for convalescents, because "they are less nutritious" than other forms.

But here again it depends entirely what kind of illness the convalescence follows. If the patient be regaining strength after pneumonia or a long wasting illness, he will need nutrition in every form of food he takes. On the other hand, if he be a gouty or plethoric person, or recovering from liver congestion or other liver disease, it may be desirable to choose for him the least nutritious diet. And it must be noted that such a recommendation is given without the least mention or concern as to what other food the patient is taking. And, of course, on this point the whole case depends. You would not need to select the most nourishing fish for a convalescent taking full supplies of meat, milk, and eggs. But if his diet for other reasons were restricted, it might then become necessary to select just that fish whose nutriment was the most. But nothing was said on this point, and many a young nurse might promptly set forth to provide her hungry convalescent with a fish whose composition afforded slight material on which to rebuild a constitution pulled down by a long and exhausting illness. It is necessary to banish once for all these baleful general rules, whose observance does so much mischief in dietetics.

The most practical plan, therefore, in nurse-dieting our patient—I use this term rather than the term of dieting, since this is the province of the physician, while "nurse-dieting" indicates the manner in which the nurse translates the doctor's orders into as many pleasant varieties of the prescribed food as it is possible to evolve—is to feed the individual as a separate person, and to consult his tastes, his fads, and his idiosyncrasies so as to suit the food to his particular necessities.

### A LADY DOCTOR IN MEXICO.

Miss Columba Rivera has been appointed as doctor to the women's ward in a Mexican hospital. She is the first woman in Mexico who has obtained such a position. She passed her medical examinations with great success.

### On Preparation for Operation in Private Houses.

By E. STANMORE BISHOP, F.R.C.S.Eng., Hon. Surgeon, Ancoats Hospital, Manchester.

AFTER undergoing a course of training in a well-recognised hospital, after seeing all the work constantly going on within its walls, after watching the work of the sisters and hearing all that they can teach you, it is no wonder if some of you imagine yourselves armed at all points and ready for any emergency. But there is one position in which many of you are certain to find yourselves at some time which at first may disconcert you not a little, because it is so different from all your previous experiences. I refer to your first engagement at a private house, and for an operation.

You are all so accustomed here to find everything adapted to the purpose—a room specially prepared and fitted for operative purposes, all the necessary apparatus and appliances specially intended for this kind of work, so accustomed to rely upon each other for help and upon your seniors for advice, that you will find it difficult to realise what your sensations will be when, for the first time, you find yourselves alone, in a strange house, with no one at hand with whom you can consult, but, rather, with everyone looking to you for advice, and, possibly enough, some with very keen eyes to detect weakness or failure on your part. After one or two experiences, of course the feeling wears off; but the first time the sensation is acute and often unpleasant enough.

Nor is it when with your own surgeons in attendance that you will be most uncomfortable. They have already seen and appreciated your work, and will make allowances for your inexperience of the new and unusual conditions in which you are placed; but other men do not know you, and may have ways of their own different to those to which you are used. You must be quick to note and accommodate yourselves to these. If you are thoroughly grounded in and understand the principles of your work, you will find no difficulty in the modification in practice preferred by one surgeon or another. Nothing, however, is more irritating to an operator than to have a nurse who can only act in one way, because it was the routine at "her hospital." And it is not only the surgeons, but the friends and relatives who have no knowledge of you, except that you are a "hospital nurse," and these have probably quite an exaggerated idea of your capabilities and the wonderful things they may expect from you. It is therefore of the utmost importance that you should have the coolness and confidence which shall satisfy them, and which is only obtained by a clear comprehension of what you have to do and the certainty of being prepared for all emergencies.

Your first care, therefore, must be to see beforehand that you have with you all the things you are likely to require, and the following list I advise you to keep by you, at all events at first: Scissors, dressing forceps, elastic catheter, enema syringe, thermometer and chart, nail brush, safety pins, vaseline. All these must be scrupulously clean, especially scissors, forceps, nail brush, and, most especially, the catheter.

Sometimes it happens that in order to have a properly trained nurse accepted as a necessity at an operation, the surgeon has to emphasise to the patient's friends their utter inability to manage some detail of the nursing, which they are often only too ready to fancy they can do amongst themselves. What we often hear is this sort of thing, "You know my wife's mother is very clever indeed in a sick-room. She has had ten children, and all the doctors round send for her if anyone is ill," &c., and I generally manage to pose them by asking if any one of them could pass a catheter if it was needed. Now if the nurse turns up without her catheter, and only finds out her omission just when it is wanted, perhaps in the middle of the night, the displaced mother-in-law, whom she might so easily have impressed by her clever manipulation, is sure, à la Mark Twain, to "see no p'ints in that frog more'n any other frog," and the nurse's position will be much more difficult, whilst the surgeon will be blamed for bringing in an incompetent or unnecessary person.

Having seen that your equipment is complete, in due time you present yourself at the house. What is your first duty after reporting yourself to the mistress? That depends to a certain extent upon the kind of domicile, whether it is a large one with many servants, or a small place where the mother or sister of your patient is the main servant of all. But generally, I should say, after making any alterations necessary in your toilette, see the patient and gain her confidence. How? It seems impertinent for me to attempt to assist you, whose sex is so much more quick in sympathy than ours. But there are some things worth remembering. All patients, however much they may try to hide it, have yet a dread of what is coming. It is more or less unknown to them, and the unknown is always the more terrifying. They look to you, whom they credit with knowing all about it, for the confidence and calmness which they lack. A pleasant, happy, confident manner sets them at their ease, for they say to themselves, "After all, things cannot be so very dreadful, or she would not look so cheerful." Little by little they will talk about this dreadful thing that is to come to-morrow; they will hint at it, skirmish round it, possibly plunge straight into the subject. Do not laugh at them or make light of it; do not attempt to even avoid the subject or evade their questions. Speak easily, calmly, kindly, and clearly to them; but do not go into details, and for heaven's sake do not be too reminiscent. It is simply dreadful for a patient to be told of this, that, or the other case that you may have seen, with all the—to them—uninteresting or horrible details, which are interesting and instructive when amongst ourselves, and to have it wound up with, "But she died, poor thing, in spite of everything." If you can mention cases that have done well in your own experience, do so by all means; but even then keep out of details. When they do not bore they only frighten or disgust your patient, and earn for you that unpleasant title, "a garrulous nurse."

If you can, as is usually the case, speak well of the operator, it is well to do so. The patient already trusts him, or she would not have chosen him for the purpose, but patients like to have their opinion confirmed by others, and it will give her the more confidence, and confidence means very much indeed in the success of most cases. Mainly, talk comfortably about her own case; that is the thing she wants to talk about, not so much to know about it as for sympathy. After all, it is what we all want, and never so much as when one is going alone, without any possible companionship, through an experience of which one knows nothing, and fears anything and everything. Picture to yourselves how delightful it must be then to feel sure of the intelligent sympathy of one who knows all about it, appreciates it thoroughly, but does not dread it, and whose kindly eyes supply us with that fortitude which we feel lacking in ourselves, which is so requisite for a successful result, and which is worth to us then more than anything on earth. But we must not let our sympathy run away with us nor detain us from the equally important duties which have to be attended to now, on the night before operation.

After seeing the patient, get on good terms with the cook. More operations are saved by the cook than by any other servant in the house, and if you can get the cook to work with you your anxieties and troubles will be immensely lessened. If there is no cook you must be cook—for the patient—yourselves. A nurse who can cook is worth twice as much as one who cannot. There is no blinking this fact, and it is sheer folly to pooh-pooh it. I know that there is a feeling in some hospitals that it is below the dignity of a professional nurse to do any cooking—even, apparently, to know when it is well done or not. Clear your minds of all that rubbish. Your *raison d'être*, as also that of the surgeon, is the cure of your patient—the restoration of her, if it is humanly possible, to a condition of health—and pride and so-called dignity, if it is in the way of this, is equally in the way of your existence as nurses, or of his as surgeon. I have seen a Fellow of the College of Surgeons, in his shirt-sleeves, make a fire and blew it into efficient action with a pair of bellows sooner than allow an operation to be done in a room where the temperature was below that of absolute safety. Yes, and he fetched the coal and sticks to do it with, too. Our whole being, our whole powers, should be, for the time, concentrated upon the one aim, the recovery of our patient, and nothing should be too great, and nothing too small, for us to do, or see to being done, to ensure that result. It is this spirit, and this alone, which will enable us to do good work as nurses or surgeons. But some may say that this is a duty which may well be left to the servants, who are there for that purpose. This is all very well if there are servants, and these servants understand invalid cookery, which most of them do not; but all operations do not take place amongst well-to-do people. There is a large class of persons who keep perhaps one servant, and perhaps none, where some member of the family—a daughter, an aunt, or a mother — undertakes this duty, and manages very well as long as nothing out of the way is required, but whose resources are completely at an end in a case like this. Often, too, in larger houses, practically the result is much the same. Cook knows how to send up a magnificent dinner, from hors d'œuvres to Gruyère, but has the haziest possible idea of what will tempt the contrary palate or be safe for the irritable stomach of her mistress after operation. So, if you desire a great reputation as a nurse, give great attention to invalid cookery; you will find it, in every sense of the word, pay. This important matter settled, run over in your mind the various things which will be required in the morning, and get everything prepared beforehand.

### Nursing Typhus in Inniskea.

Hospital nursing has its drawbacks, and district work its difficulties, but there is something unique about the drawbacks and difficulties that encounter one when nursing in the west of Ireland.

The Inniskea Islands, lying off the coast of Mayo, have lately been the centre of a severe outbreak of typhus. The fever spread rapidly among the half-starved, over-crowded inhabitants. Of what avail were Atlantic breezes when in a two-roomed cottage there resided ten human beings, three cows, three pigs, and innumerable fowl? But this was a superior cottage, owned by a family who could boast a double and hyphened name. The island cottages more often consist of a single room—at once bed-sitting-room, kitchen, and farmyard. The chickens roost on the bed-posts, the cow is usually in a corner with her head to the wall, a hole in the roof acts as chimney when there is sufficient draught to draw the smoke up, and a hole in the floor answers as dustbin and cesspool. Occasionally for æsthetic purposes this latter hole is covered with a paving stone. Large stones support the front door, since hinges are regarded as a modern innovation; back doors and windows are conspicuous by their absence; where they exist they are carefully constructed so as never to open.

When, after a drive of sixty-three miles from the nearest railway terminus and ten miles in an open boat, two nurses from the City of Dublin Nursing Institute (Miss Kenny and Miss Macalister) landed on the South Island, the inhabitants flocked to the shore to meet them. The fishermen, who can almost all speak English, welcomed them in that tongue, while their wives and mothers blessed the nurses in Irish. But all this happened long ago, on June 9th, 1897, before the islanders knew that these nurses—in fact, all trained nurses—are the sworn foes of dirt, parasites, and superstition, and the devotees of such strange gods as fresh air, soap, and water.

Then came the long struggle with the gods of the island, a struggle which is not yet over. The doctor, nurses, and parish priest all trying to persuade the Inniskeaites that though their fathers may have gone to bed with all their clothes on and refused to be washed till the fever was over, yet that in this nineteenth century science had discovered surer and better methods of treating typhus. The traditions of the island and the innate good nature of the inhabitants rather increased the difficulties of nursing. Men and women who knew what it was to be hungry could not bear to see poultices wasted on pneumonia patients, when the linseed might make such a good meal for the starved pigs.

In health, the Inniskeaite is devoted to whisky, and his beloved "poteen," but according to some unwritten island law, in fever only "cauld water" must be drunk till "the cool," when the patient is supposed to have recovered. What is to be done when the friends and relatives of a man in a severe rigor absolutely refuse to let him be given a drop of stimulant, for "shure an' he's dying, and let him go to his God without the smell of the dthrink"?

On the whole the death-rate has been very low. The islanders seem to have wonderful vitality, but little or no recuperative power. They will not die, but neither will they recover—no doubt this debility is due to the starved condition of the inhabitants. When potatoes can be obtained they enter largely into the island dietary, but apparently the staple food is Indian meal; eggs, milk, dried fish, and oatmeal are used when they can be had.

As soon as the condition of the islanders became known, food and fuel were sent over from the mainland, and not the least arduous part of the nurses' work was distributing the supplies. Sometimes both doctor and provisions would be weather-bound, no fishing boat venturing to put out for days, or the meal would arrive caked together with salt water.

When Miss Kenny and Miss Macalister finally themselves got fever, and had to be brought to Belmullet Hospital, they crossed, with temperatures between 104 degs. and 105 degs. Fahr., in open boats, over which the waves were washing.

In a few short weeks, however, wonders have been worked by the Local Government Board officials. The picturesquely dirty cottages of Inniskea, with their thatched roofs of waving oats and weeds, have been converted into a white-washed village, and H.M.S. *Albacore* replaces the national "Curragh."

Two hospitals in the care of a resident doctor and five nurses have been started; when the doctor and three nurses contracted typhus, another doctor and new nurses took their place. And these things the Local Government Board officials have done, though they must have known that "Shure 'twas the will o' God the faver should rage." Still worse, they compel the fever-stricken islander, not only to enter the hospital, but to submit to being washed and lying between clean sheets!

As yet fire from Heaven has not consumed the representatives of the Local Government Board in the West. Nay, rather the fever seems to be abating, and elated by apparent success, they have put the Public Health Acts into force, and actually declare a man may not keep his own pig under his own bed!

### The Nursing of Beri-Beri.

From a Nurse Correspondent.

The outbreak of so mysterious and unusual a disease as beri-beri has made the Richmond Asylum, Dublin, most interesting to doctors and nurses. The asylum has long had a somewhat unenviable reputation from the hygienic point of view, so that I was agreeably surprised to find it comparatively light, bright, and cheerful, though undoubtedly most overcrowded. Altogether there are about 170 patients in all stages of the disease, and Dr. Connoly Norman most kindly and courteously took me round and explained the various stages through which the disease passes, with its care and nursing. One of the first patients visited was a nurse, now up and sufficiently convalescent to be thoroughly enjoying a copy of The Hospital. Seven nurses altogether have been attacked by the horrible disease, with all its chronic after effects. And there could hardly be a finer tribute to the loyalty and devotion of a nursing staff than the fact that no nurse has left the asylum on account of the prevalence of beri-beri since 1894, when the epidemic first showed itself. I saw two or three nurses who were attacked by the disease during the second epidemic in 1896, and who are so unfortunate as to have taken it again. It appears that one attack predisposes to a second.

Dr. Norman says beri-beri invariably leaves heart irritability, in some cases going on to dilatation and disease, death frequently taking place from the weakness of the heart action. The mortality during this epidemic has been very slight indeed, for experience has taught the staff to recognise the disease and take precautionary measures in the early stages. In 1894, for some time, the disease was not diagnosed, for beri-beri would not be looked for in a European hospital. But the attention of the staff was soon directed to the fact that many of the patients were showing symptoms of paralysis and dropsy. And it appears that the association of these two symptoms is met with only in beri-beri. In milder cases paralysis is only partial, and the loss of sensation in the limbs not quite complete. In other types there is extreme tenderness with dropsical pitting. A few patients were affected with extreme squinting, and in two or three suppuration had appeared on the limbs. Many of the worst cases were those who had never recovered from the outbreak of last year. Anæmia, weakness, and prostration were very marked in some cases, many of the patients being quite unable to stand up, and in attempting to walk the movements of the limbs became quite choreic. Galvanic batteries were being freely used to test the areas of sensibility in the limbs, and very elaborate charts of human figures were being made, the outlines of returned sensibility being shown in red pencil. From a nursing point of view these charts were most interesting. I was very glad to find that the Richmond Asylum employs only trained women nurses, both in the male and female wards of the infirmary, for I think this is a point wherein our English asylums fall short of the standard which should obtain. And I was much impressed by the kindness shown to the patients by the nurses. Insane persons are at all times very trying, but when their condition is aggravated by so distressing a disease as beri-beri, the care of such patients is no light tax on patience and kindness. The wards are most clean, bright, and light, decorated with flowers; the bedding is much coarser than ordinarily used in England, but beautifully clean. The nursing consists chiefly in good care and feeding, guarding against chills, and treating the cases more or less as heart cases. Fortunately, beri-beri does not appear to increase the mental trouble of the patients—indeed, it doubtless has the effect of rendering them more apathetic and passive. The extreme pallor and exhaustion of the beri-beri patients is very marked. The asylum grounds are very large, and the position it occupies is high, and should be healthy. But in the wards the beds are much too close together for hygienic requirements, although the ventilation seemed excellent. It was most pleasant to see the affectionate enthusiasm with which Dr. Norman was received during our progress through the wards. The most pathetic among the cases were the foreign patients—Italian and French governesses—whose reason had become affected while in Ireland, and whose friendlessness caused them to drift into a public asylum. Some of these poor women speak no language but their own, thus cutting them off from the consolation of exchanging ideas with fellow patients. To these especially Dr. Norman's round seemed particularly grateful, for he never failed to stop a moment and have a short conversation in their own tongue with these unfortunate women. The neighbouring prison of Grangegorman is to be emptied of its prisoners, and the building—a very healthy one—used to help to thin out the surplus asylum inmates. This step, with the thorough disinfection and cleansing which will be carried out as soon as some of the asylum wards can be cleared, it is hoped will have the effect of banishing beri-beri from Dublin, even as St. Patrick banished snakes from Ireland.

### The Man with a Brougham.

We have in the past heard complaints from nurses as to their treatment by the authorities of nursing institutions. The criminal classes are known to have a tender place in their hearts for doctors and nurses, and, although the nurse suffered most in the end, it is possible to conceive that the "man with a brougham" originally intended to take it out of the Wigmore Institute rather than the nurse. Probably the rogue argued that, as he was introduced to the nurse by the superintendent of the institute, the latter will *de facto* have to compensate the nurse and bear the loss. But to the story. A man with a brougham drove up to the Wigmore Institute in Weymouth Street, interviewed Miss Fulorence Burell, the principal, mentioned the name of Dr. Crisp, of Tooting Common, and induced her to take him to 81, Marylebone Street, where he was introduced to and engaged the services of Nurse Trusler for an urgent case, to which he would drive her in the brougham. He first stole the nurse's portmanteau by taking it to Portland Road Station whilst the nurse was dressing because his master objected to luggage being placed in his carriage. This done he put the nurse inside the brougham, and then spent an hour or so in driving to a chemist's in Sloane Street, then to St. James's Street, where they waited twenty minutes, and then to Adelphi Chambers, Strand. Here the man borrowed ten shillings from the nurse, and when she ventured to point out that "the urgent case" was being neglected, and that the doctor would be angry, he drove her to "Apothecaries' Hall, in Berners Street" to fetch a set of surgical instruments. Berners Street is near Portland Road Station, and possibly that is why he asked the nurse to take a little light refreshment in an A.B.C. shop whilst he walked down Oxford Street. In any case the man disappeared, and after waiting for two more hours the nurse took counsel with the driver, visited Dr. Crisp's at Tooting, and found that this man with a brougham had borrowed seven more shillings from the driver, deceived Mrs. Crisp, and duped the livery stable keeper. The goods are stated to be so marked as to be easily identifiable; so the man may yet be laid by the heels, despite his brilliant abilities as already related. Meanwhile we have small doubt that every private nurse will for the future keep a sharp eye upon all visitors being men with broughams.

### Beverages for Invalids.

ALMOND MILK.—Great care must be taken to make and keep this in a cool place, as heat will turn it sour. Put one ounce of best Jordan almonds and a quarter of an ounce of bitter ones into a stewpan and cover with cold water; put it on the stove, and as soon as they boil rinse them in cold water, and rub the almonds in a clean cloth, remove the skins, and pound the nuts in a mortar with a dessert spoonful of orange-flower water, and one ounce of castor sugar; add a few drops of water, and pound altogether till the paste is smooth. Put in a clean basin, and add half a pint of clear water (cold), stirring it with a silver spoon. After it has stood for two hours, strain and serve with an equal quantity of water added to the almond milk.

ORANGE ICE.—Pound sufficient ice pounded very small to fill half a tumbler, put in a teaspoonful of castor sugar and the strained juice of a good orange; mix altogether and serve at once.

APPLE WATER.—Put four baked apples into a jug, sprinkle over them a dessert spoonful of castor sugar, add one pint of boiling water, cover the jug, and when quite cold strain off and use the same day. This will not keep long without fermenting.

EGG WINE.—Mix the egg well with about a tablespoonful of cold water, half a teaspoonful of castor sugar, and a tiny dust of nutmeg; pour on to this a quarter of a pint of hot water, in which put one glass of the best sherry (if allowed by the doctor). Strain into a clean glass.

NARISSA MILK.—Mix two teaspoonfuls of Narissa to a paste with a little cold milk. Pour upon it half a pint of boiling milk, stirring it well; return to a clean stewpan and boil for five minutes; add two lumps of sugar, and strain into a cup before serving.

COFFEE.—To make good coffee, two ounces that has been freshly ground must be allowed to a pint of water. Put the coffee into a percolator and pour the boiling water slowly over it; or put the coffee into a clean stewpan, mix with a dessertspoonful of cold water and the white of a small egg. Pour the boiling water on them, and let it come to the boil for one minute; strain through a fine strainer, and serve with boiling milk and a little cream.

COCOA.—The making of cocoa depends upon the kind used. Cocoa nibs require to be stewed for several hours, and those that have starch in them must also be boiled, but only for a short time. Preparations of cocoa that have no starch in them will be sufficiently cooked by having boiling water poured on them.

RICE MILK.—Put two ounces of Carolina rice into a stew-pan and cover with cold water, add a pinch of salt and bring to the boil; then wash it in cold water and put it into one pint of boiling milk and let it simmer for ten minutes; strain off, and add sugar or salt to taste.

LEMONADE.—Pare the rinds of four lemons as thinly as possible; put them into a jug and squeeze the juice of the lemons through a strainer on to them; add half a pound of lump sugar and a quart of cold water, let it stand for three hours, then pass the lemonade through a jelly bag, add more water if necessary, and the raw whites of two eggs.

BARLEY WATER.—Well wash two ounces of pearl barley in cold water; put it in a jug with the peel of a lemon cut very thinly, and six lumps of sugar. Pour one quart of boiling water on them, cover the jug closely, and when quite cold pour off the barley water into a clean glass jug.

TOAST AND WATER.—Cut a hard crust from a stale loaf of bread and toast it till it is a nice dark brown, but be very careful not to burn it. Put it in a jug and pour one quart of boiling water on it, cover it tightly, and let it get quite cold before straining it for use.

CELERY TEA FOR RHEUMATISM.—Clean two heads of celery, removing the green leaves; cut up the celery, and put it into a clean stewpan; add two quarts of cold water, bring it quickly to the boil, then simmer gently for one hour; put it altogether into a large jug, and when quite cold strain off and use.

### The Book World for Women and Nurses.

THE MIDWIVES' POCKET BOOK. The Burdett Series, No. 3. By HONNOR MORTEN. (London: Scientific Press. 1897. Price, 1s.)

This is a handy little book, which will serve to remind the midwife of many things which she has learned during her training, and thus give her confidence in her work. Strict limitations are placed on the sort of work which a midwife may do. This, however, is no disadvantage. It is stated that a midwife must be competent to attend a natural labour, that she should easily diagnose abnormality at an early stage, and that in abnormal labour it is her duty to send for a physician. A list of training schools is given, a good many details are also entered into as to the preparation for the L.O.S. examination, and these are followed by a description of the examination itself and a list of the certificates required. Samples are then given of the papers set at various examinations, after which come a couple of chapters on anatomical details, and on the positions of the fœtus, and a chapter giving the rules for conducting a case, the remainder of the book being occupied with a "Practical Alphabetical Guide," in which many useful details are alphabetically arranged. As we have said, it will be found a handy little book for a midwife to have in her bag, but it must be understood that it does not pretend to be a complete manual. The real teaching of midwifery must be got at the training school, not from books.

### Minor Appointments.

SANATORIUM FOR CONSUMPTION, BOURNEMOUTH.—Miss Nina M. Armstrong has been appointed Charge Nurse at this institution. She was trained at the Croydon General Hospital and the Yeovil District Hospital, and has been staff nurse at the Croydon Hospital and assistant nurse at the Surrey Convalescent Home.

UNION INFIRMARY, STRATFORD-ON-AVON.—Mrs. Alice Fleetwood has been appointed Charge Nurse at the above infirmary. She was trained at the Mill Road Infirmary, Liverpool, and has held the appointment of night nurse at the Coventry Infirmary Union.

BATH UNION INFIRMARY.—Miss Charlotte Machin has been appointed Charge Nurse to the above infirmary. She received her training and worked for four and a-half years at the Sherburn Hospital, County Durham. Miss Machin has excellent testimonials.

UNION INFIRMARY, SUNDERLAND.—Miss Elizabeth McCoy has been appointed Night Charge Nurse of the above institution. She has been previously employed at the Fir Vale Infirmary, Sheffield.

POPLAR AND STEPNEY SICK ASYLUM.—The vacant post of Sister at the above institution has been filled by Miss H. W. Brown, who was trained for three years at the Brownlow Hill Infirmary, Liverpool.

WIMBORNE AND CAMBORNE UNION.—Miss Eleanor Kewish has been appointed Charge Nurse at the above institution. She was trained at the Crumpsall Infirmary, and has since been employed at the Runcorn Union.

### Novelties for Nurses.

### A CONVENIENT DRINKING CUP.

The question of bulk always arises when impromptu picnicing is proposed, and on this score an economical proposal to picnic when touring is often set aside. The Unique Manufacturing Company, whose headquarters are at Rotherham, has sent us a specimen of so neat a contrivance —which they call the Jubilee Pocket Drinking Cup—that a cup of tea is now placed within the reach of all wanderers abroad who have been daunted heretofore by other non-compressible cups. The little cup is made of a tough paper like leather in appearance, which folds quite flat and takes no appreciable room. The manufacturers do not claim for it that it can be used as a tea cup, but we have tested for ourselves and find it is quite convenient in that capacity, and owing to its cheapness it can without hesitation be discarded after use. When used for cold drinks and when immediate rinsing is possible the cup can have a longer life. Anyhow it is a most excellent little contrivance, and with it a small heating tin containing ready-made tea and a small spirit lamp, a portable tea equipage of the compactest description is available for the traveller whether on foot, bicycle, in the train, or otherwise.

### RED CROSS NIGHT LIGHTS.

Night lights are a necessity in the sick-room, and, curiously enough, they are even more necessary now than when candles or rush lights were in use. By universal consent, it is agreed that gas should not be kept burning through the night in the sick chamber, and, as for the electric light, unless some very special arrangements are made, the sudden turning on of the full glare is enough to rouse many patients, and especially children, to such a condition of wakefulness that a return of sleep may be long in coming. Night lights, then, are essential, and we are able very confidently to recommend those prepared by Messrs. Francis Tucker and Co., High Street, Kensington, and sold under the name of Red Cross Night Lights. Not only do they give a steady light, but no water is required, and there is complete freedom from smoke and smell. They are, moreover, a very safe light. The wick is stamped into a metal holder, so that it is impossible for it to fall, with the resulting advantage that it burns to the end; it cannot either flare or smoke, or crack the glass. A couple of the glasses are supplied with each box of Red Cross Night Lights, which are altogether worthy of recommendation.

### Presentations.

DEWSBURY GENERAL INFIRMARY.—The matron at the Dewsbury and District General Infirmary (Miss Nunn) has delivered a course of lectures on sick nursing, &c., to the members of the Heckmondwike Industrial Co-operative Society's Women's Guild during the past six months. At the close of one of these lectures on Tuesday evening, the president (Miss Wormald) presented to Miss Nunn, on behalf of the members of the guild, a valuable gold-mounted umbrella. In making the presentation, Miss Wormald spoke in eulogistic terms of the lectures, and said all the members had been greatly benefited by them. Miss Nunn suitably acknowledged the presentation.

MISS AMELIA HOLBROOK was presented with a handsome writing case by her fellow-nurses of the Union Infirmary, Sunderland, on her departure to take up the post of night superintendent of the Union Infirmary, King's Norton.

### Everybody's Opinion.

[Correspondence on all subjects is invited, but we cannot in any way be responsible for the opinions expressed by our correspondents. No communication can be entertained if the name and address of the correspondent is not given, or unless one side of the paper only is written on.]

### ASYLUM TRAINING.

DR. HERBERT BARRACLOUGH writes: Would you kindly allow me to reply as briefly as possible to your criticism of the letter I wrote to the *British Medical Journal*? The one point to which you take exception is as to the practical training for the Medico-Psychological Nursing Certificate. Well, sir, more and more is the practical side of this training being laid stress upon, both by medical officers and examiners. The general rule is to have at least one practical class a week, and in some instances two of these classes are held. In this asylum entrance for the examination is not compulsory, and yet I find that nurses who have no intention of taking the certificate attend the practical part of the work and enter into it with the most exemplary enthusiasm. And they are taught all that comes into the domain of sick nursing. With regard to experience in a sick ward, as far as possible all nurses serve for a time in our hospital, and by the time they are eligible for the post of charge nurse they have a very fair experience of nursing the sick within the necessary limits of asylum work. Pressure upon your space forbids my saying more, save to plead most earnestly that a little more sympathy may be extended by our professional brethren towards those of us who are striving to raise the standard of mental nursing.

[Dr. Barraclough evidently does not appreciate the difference which lies in training by means of a practical class and that secured by constant attendance by day and night upon the sick for a considerable period.—ED. *T. H.*]

### Where to Go.

MIDWIVES' INSTITUTE.—The following lectures will be held at the institute in October: Monday, October 25th, at three o'clock, Mrs. Hewer will lecture on "The Duties of a Monthly Nurse before the Confinement." Admission, 2s. The midwifery lectures are on the 25th to 28th, at four o'clock. Subjects, "Pelvis and Fœtal sense," and "The Organs of Generation." Admission to a single lecture, 2s. 6d. Mrs. Scharlieb, M.D., B.S., will lecture on "The Diagnosis of Swellings Behind the Uterus" at the Midwives' Institute, 12, Buckingham Street, Strand, on Friday, October 22nd, at 7.45. A few tickets of admission can be obtained for non-members, 6d. each. Preparations for Obstetric Society of London examination for midwives in January commences October 25th. All particulars can be obtained from the secretary at the club. Fee for the course, £3 3s.

### Appointments.

### MATRONS.

BIRMINGHAM AND MIDLAND HOSPITAL FOR WOMEN.—The vacancy of Matron at this institution has now been filled by the appointment of Miss Kate Eleanor Richmond. Miss Richmond was trained at the Edinburgh Royal Infirmary, has been charge nurse at the Hartlepool Infirmary, assistant sister at the Paddington Infirmary, and assistant matron at the Midland Counties Home for Incurables.

KETTERING AND DISTRICT GENERAL HOSPITAL.—Miss Gertrude Hick has been appointed Matron to the above hospital. She has hitherto held the post of matron at the Oriolet Cottage Hospital, Loughton, and received he training at the London Hospital.

### For Reading to the Sick.

"COMMITTING OUR WAY TO GOD.'

**Verses.**

GOD does not need
Either man's works, or His own gifts; who best
Bears His mild yoke, they serve Him best. His state
Is kingly; thousands at His bidding speed,
And post o'er land and ocean without rest;
They also serve who only stand and wait. —*Milton.*

Though some good things of lower worth
My heart be called on to resign,
Of all the gifts in heaven and earth
The best, the very best is mine,
The love of God in Christ made known—
The love that is enough alone—
My Father's love is all my own.

My Soul's Restorer, let me learn
In that deep love to live and rest;
Let me the precious thing discern
Of which I am indeed possess'd.
My treasure let me feel and see,
And let my moments as they flee
Unfold my endless life in Thee.

—*Hymns and Meditations.*

There is a secret in the ways of God
With His own children, which none others know,
That sweetens all He does. —*Swaine.*

**Reading.**

With what perfect and entire confidingness did Jesus commit Himself to His Heavenly Father's guidance. He loved to call Him "My Father." There was music in that name, which enabled Him to face the most trying hour, to drink the most bitter cup. How many a perplexity should we save ourselves by thus implicitly "committing ourselves," as He did, to the Righteous Judge. In seasons of darkness and trouble, when our way is shut up with thorns, to lift the confiding eye of faith to Him and say, "I am oppressed, undertake for me." How blessed to feel that He directs all that befalls us, that no contingencies can frustrate His plans; that the way He leads is not only *a* right way, but with all its briars and thorns, its tears and trials, it is *the* right way! Look back on your chequered path. How wondrously has He threaded you through the mazy way. . . . No affliction will be sent you greater than you can bear. His voice will be heard stealing from the bosom of the threatening cloud, "Be still, and know that I am God."—*From "The Words of Jesus."*

Be patient! Beware of hastiness of speech or temper; remember how much evil may be done by a few inconsiderate words "spoken unadvisedly with the lip." Think of Jesus standing before a human tribunal, in the silent submissiveness of conscious innocence and integrity. Leave thy cause with God. Let this be the only form of complaint, "O God, I am oppressed; undertake Thou for me!" "In patience," then, "possess ye your souls." Let it not be a grace of peculiar seasons, called forth on peculiar exigencies; but an habitual frame manifested in the calm serenity of a daily walk—placidly amid the little petty annoyances of everyday life—a fixed purpose of the heart to wait upon God and cast its every burden upon Him.

—*"The Words of Jesus."*

### Notes and Queries.

**The contents of the Editor's Letter-box have now reached such unwieldy proportions that it has become necessary to establish a hard and fast rule regarding Answers to Correspondents. In future, all questions requiring replies will continue to be answered in this column without any fee. If an answer is required by letter, a fee of half-a-crown must be enclosed with the note containing the enquiry. We are always pleased to help our numerous correspondents to the fullest extent, and we can trust them to sympathise in the overwhelming amount of writing which makes the new rules a necessity. Every communication must be accompanied by the writer's name and address, otherwise it will receive no attention.**

*Workhouse Nursing.*

(1) Would training in a workhouse infirmary be as efficient as in a hospital, and could I obtain a hospital appointment afterwards?—*Alice.*

In some of the very large workhouse infirmaries, such as that at Birmingham, you would secure admirable training, but, as a rule, there is not enough variety in the cases to afford such good practice as in a hospital. You would not find it easy to obtain an appointment at a hospital if trained at a small workhouse infirmary.

*Spirits of Salt.*

(2) Where can I obtain dulcified spirits of salt?—*Dorothy.*

This can be obtained at a chemist's.

*Holt-Ockley System.*

(3) What is the Holt-Ockley system of nursing? How long are the nurses trained for?—*Annie.*

You will find particulars of this scheme stated and discussed in THE HOSPITAL Nursing Mirror for October 5th, 1895. The training is for six months, but the candidate must have had some preliminary experience.

*Questions of Etiquette.*

(4) Is it etiquette for a lady nurse on social visiting terms with the doctor for whom she is nursing to bow to him on meeting him in the street when in uniform, or should she wait for him to bow first? Would uniform or ordinary dress make any difference? What is the etiquette on this point when the nurse only knows the doctor professionally?—*Verona.*

Good manners are always etiquette and suitable. It is a lady's privilege, in whatsoever dress she may be, to bow first to her masculine acquaintance. If she does not it expresses her wish to "cut" him. Naturally, therefore, a doctor would not take the initiative, as he might be "snubbed" for his pains. Why should a professional acquaintance require a different ruling to a social one? A bow is the simplest form of recognition and suitable in every case.

*Probation at 40.*

(5) 1. Will you kindly tell me if there is any hospital in London where probationers at the age of 40 are received for training? 2. Is there any branch of nursing that can be taken up by one of that age, who is naturally cheerful, active, energetic, and in good health?—*Ellen Dorrett.*

1. None that we know of. Thirty-five seems the general age limit. 2. You seem excellently qualified for midwifery. The General Hospital, York Road, offers first-rate training. The course occupies about six months for a London Obstetric Society's certificate, and the cost is not heavy. If you think well of this suggestion write direct to the matron.

*Probation for a Queen's Nurse.*

(6) 1. Are probationers without previous experience permitted to join for training the Queen Victoria Jubilee Institute? 2. Do they receive a salary?

No name and address accompanies this query; therefore, by our rules we cannot answer it.

*The "Organ" of "a Profession."*

(7) Will you kindly inform me whether THE HOSPITAL is the recognised organ of the nursing profession? If not, perhaps you would kindly tell me which is the journal which can claim this title, and oblige. *Ignoramus.*

The boundaries of the "nursing profession" are so ill-defined that the above question in the form in which it is put does not seem to admit of a direct answer. If, however, we were asked, Which is the best or most widely recognised organ of the "nursing profession" we could have little hesitation in naming THE HOSPITAL Nursing Mirror.

*Peptonised Milk.*

(8) Will you tell me how to peptonise milk, and also of a text-book containing the information?—*G. B.*

One method of peptonising milk is to dilute a pint of milk with a quarter of a pint of water and heat to a temperature of 140 degrees. Then mix with the hot milk two teaspoonfuls of liquor pancreaticus and twenty grains of bicarbonate of sodium. The mixture is then poured into a covered jar and placed in a warm place to keep up the heat. At the end of an hour and a half the milk is raised to the boiling point for a few seconds, after which it can be used as ordinary milk. This and another recipe are to be found in "Diet in Sickness and in Health," by Mrs. Ernest Hart (London: The Scientific Press, 28 & 29, Southampton Street, Strand).

*Army Nursing.*

(9) Would training at the City Hospital, Birmingham, and the Seamen's Hospital, Greenwich, qualify me for the post of Army Sister? —*M. C.*

You are not likely to be selected unless you have had at least three years' training in one large general hospital.

